# Supplementary material for: Hot spot prediction in protein-protein interactions by an ensemble system
Source: BMC Syst Biol. 2018 Dec 31;12(Suppl 9):132. doi: 10.1186/s12918-018-0665-8 (PMC6311905; doi:10.1186/s12918-018-0665-8)
Supplement: Supplementary file 2 — All relevant MATLAB code. A simple Matlab implement of our predictor. (ZIP 19,742 kb) [file 12918_2018_665_MOESM2_ESM.zip › Additional file 2/Read ASA/ASA website/dbMPIKT/5CYKA.html]

 NetSurfP - Protein Surface Accessibility and Secondary Structure Predictions

  

|  |  |  |
| --- | --- | --- |
|  |  | NetSurfP - Protein Surface Accessibility and Secondary Structure Predictions  Technical University of Denmark |

  


---

```
# For publication of results, please cite:
# A generic method for assignment of reliability scores applied to solvent accessibility predictions.
# Bent Petersen, Thomas Nordahl Petersen, Pernille Andersen, Morten Nielsen and Claus Lundegaard
# BMC Structural Biology 2009, 9:51 doi:10.1186/1472-6807-9-51
#
# Column 1: Class assignment - B for buried or E for Exposed - Threshold: 25% exposure, but not based on RSA
# Column 2: Amino acid
# Column 3: Sequence name
# Column 4: Amino acid number
# Column 5: Relative Surface Accessibility - RSA
# Column 6: Absolute Surface Accessibility
# Column 7: Z-fit score for RSA prediction
# Column 8: Probability for Alpha-Helix
# Column 9: Probability for Beta-strand
# Column 10: Probability for Coil
E M  5CYKA                  1    0.667 133.487  -1.992   0.003   0.003   0.994
E D  5CYKA                  2    0.727 104.703  -0.421   0.018   0.047   0.935
E A  5CYKA                  3    0.503  55.409  -0.773   0.053   0.043   0.903
E P  5CYKA                  4    0.630  89.454   0.760   0.052   0.084   0.864
E M  5CYKA                  5    0.390  78.099  -1.031   0.056   0.142   0.802
E E  5CYKA                  6    0.566  98.880  -0.957   0.052   0.084   0.864
E D  5CYKA                  7    0.734 105.798  -0.347   0.052   0.084   0.864
E A  5CYKA                  8    0.428  47.122  -0.614   0.052   0.084   0.864
E P  5CYKA                  9    0.673  95.428  -0.560   0.018   0.047   0.935
E A  5CYKA                 10    0.512  56.477  -0.375   0.018   0.047   0.935
E P  5CYKA                 11    0.589  83.636  -0.505   0.018   0.047   0.935
E V  5CYKA                 12    0.551  84.719  -0.374   0.004   0.085   0.910
E A  5CYKA                 13    0.268  29.490  -0.331   0.005   0.336   0.660
E Q  5CYKA                 14    0.381  68.118   1.172   0.001   0.900   0.099
B V  5CYKA                 15    0.044   6.747   1.199   0.001   0.959   0.040
B K  5CYKA                 16    0.180  37.108   1.686   0.001   0.959   0.040
B V  5CYKA                 17    0.023   3.612   1.017   0.001   0.959   0.040
B I  5CYKA                 18    0.136  25.234   0.980   0.001   0.959   0.040
B F  5CYKA                 19    0.030   5.961  -0.195   0.001   0.900   0.099
B T  5CYKA                 20    0.111  15.340   0.938   0.004   0.616   0.381
B T  5CYKA                 21    0.175  24.259  -0.141   0.004   0.138   0.858
E T  5CYKA                 22    0.616  85.425  -0.068   0.003   0.003   0.994
E E  5CYKA                 23    0.430  75.069  -1.741   0.016   0.005   0.979
E P  5CYKA                 24    0.583  82.742  -0.139   0.018   0.088   0.893
E D  5CYKA                 25    0.367  52.914  -0.542   0.021   0.451   0.528
B L  5CYKA                 26    0.129  23.693   0.164   0.023   0.655   0.322
B E  5CYKA                 27    0.188  32.826  -1.030   0.023   0.655   0.322
B L  5CYKA                 28    0.126  23.071  -0.633   0.021   0.451   0.528
B P  5CYKA                 29    0.274  38.895  -1.099   0.005   0.262   0.733
E E  5CYKA                 30    0.395  69.076  -1.404   0.004   0.085   0.910
E S  5CYKA                 31    0.588  68.960  -1.814   0.005   0.015   0.979
E K  5CYKA                 32    0.664 136.564  -1.162   0.018   0.019   0.964
E R  5CYKA                 33    0.402  92.058  -0.563   0.004   0.085   0.910
E Q  5CYKA                 34    0.293  52.312  -0.056   0.004   0.616   0.381
B L  5CYKA                 35    0.064  11.755  -0.113   0.001   0.900   0.099
E L  5CYKA                 36    0.247  45.299   0.468   0.001   0.900   0.099
B V  5CYKA                 37    0.086  13.157   0.921   0.001   0.900   0.099
B P  5CYKA                 38    0.213  30.281  -0.612   0.002   0.816   0.182
B A  5CYKA                 39    0.133  14.646  -0.461   0.023   0.655   0.322
B D  5CYKA                 40    0.247  35.636  -0.836   0.022   0.552   0.426
B I  5CYKA                 41    0.056  10.286   0.569   0.022   0.552   0.426
E R  5CYKA                 42    0.338  77.310   1.056   0.022   0.552   0.426
B R  5CYKA                 43    0.193  44.174  -0.194   0.074   0.484   0.442
E Y  5CYKA                 44    0.322  68.811  -0.114   0.191   0.086   0.723
E G  5CYKA                 45    0.574  45.190   0.259   0.257   0.016   0.727
B L  5CYKA                 46    0.165  30.230  -0.722   0.622   0.015   0.363
E S  5CYKA                 47    0.479  56.080  -0.446   0.660   0.049   0.291
E R  5CYKA                 48    0.494 113.172   1.434   0.701   0.107   0.192
B I  5CYKA                 49    0.109  20.165   0.414   0.725   0.163   0.112
B L  5CYKA                 50    0.126  23.126   0.476   0.578   0.229   0.194
E N  5CYKA                 51    0.458  67.095   0.958   0.478   0.309   0.214
E S  5CYKA                 52    0.379  44.442   1.426   0.373   0.401   0.226
B E  5CYKA                 53    0.072  12.543   0.345   0.252   0.423   0.325
E S  5CYKA                 54    0.495  58.026   0.747   0.135   0.317   0.548
E M  5CYKA                 55    0.459  91.826  -0.963   0.052   0.084   0.864
B L  5CYKA                 56    0.200  36.620  -0.240   0.018   0.047   0.935
E D  5CYKA                 57    0.697 100.510  -0.343   0.018   0.019   0.964
E T  5CYKA                 58    0.503  69.780  -1.024   0.005   0.015   0.979
E G  5CYKA                 59    0.385  30.284   0.775   0.004   0.085   0.910
B S  5CYKA                 60    0.034   3.997  -0.630   0.004   0.616   0.381
B I  5CYKA                 61    0.172  31.746   1.169   0.001   0.900   0.099
B P  5CYKA                 62    0.035   4.995  -0.545   0.001   0.900   0.099
B F  5CYKA                 63    0.023   4.516   1.074   0.001   0.959   0.040
B D  5CYKA                 64    0.085  12.234  -0.114   0.001   0.959   0.040
B F  5CYKA                 65    0.040   7.948   0.180   0.001   0.900   0.099
B L  5CYKA                 66    0.088  16.040   0.660   0.004   0.197   0.799
E I  5CYKA                 67    0.416  76.979  -0.084   0.005   0.045   0.951
E N  5CYKA                 68    0.547  80.081   0.214   0.003   0.003   0.994
E G  5CYKA                 69    0.260  20.470  -0.506   0.016   0.005   0.979
E S  5CYKA                 70    0.443  51.943   1.170   0.018   0.088   0.893
B F  5CYKA                 71    0.172  34.420   1.798   0.018   0.846   0.136
B L  5CYKA                 72    0.018   3.332   1.536   0.011   0.918   0.071
B R  5CYKA                 73    0.046  10.465   0.505   0.011   0.918   0.071
B S  5CYKA                 74    0.027   3.118  -0.417   0.018   0.846   0.136
B S  5CYKA                 75    0.032   3.727  -0.788   0.004   0.616   0.381
B L  5CYKA                 76    0.041   7.525  -1.805   0.005   0.336   0.660
E E  5CYKA                 77    0.256  44.758  -0.465   0.018   0.047   0.935
B D  5CYKA                 78    0.196  28.244  -1.259   0.018   0.047   0.935
E Y  5CYKA                 79    0.291  62.187   0.136   0.019   0.141   0.840
B L  5CYKA                 80    0.043   7.818  -0.306   0.022   0.552   0.426
B T  5CYKA                 81    0.046   6.422   0.488   0.011   0.918   0.071
B S  5CYKA                 82    0.138  16.209   0.707   0.011   0.918   0.071
B N  5CYKA                 83    0.115  16.865   0.953   0.011   0.918   0.071
B G  5CYKA                 84    0.093   7.319   0.066   0.018   0.846   0.136
B L  5CYKA                 85    0.113  20.690   0.463   0.002   0.816   0.182
E S  5CYKA                 86    0.333  39.051   0.011   0.004   0.616   0.381
B L  5CYKA                 87    0.151  27.557  -0.318   0.019   0.141   0.840
E E  5CYKA                 88    0.707 123.600   0.384   0.018   0.088   0.893
E T  5CYKA                 89    0.579  80.280   0.518   0.018   0.047   0.935
B T  5CYKA                 90    0.265  36.728  -0.728   0.019   0.141   0.840
E L  5CYKA                 91    0.548 100.430   0.574   0.004   0.514   0.481
E T  5CYKA                 92    0.423  58.684   1.091   0.002   0.816   0.182
E L  5CYKA                 93    0.236  43.230   1.312   0.001   0.900   0.099
E Q  5CYKA                 94    0.286  51.097   0.952   0.001   0.959   0.040
B Y  5CYKA                 95    0.260  55.519   1.119   0.001   0.959   0.040
B V  5CYKA                 96    0.061   9.330   0.408   0.001   0.900   0.099
E R  5CYKA                 97    0.555 127.003   0.408   0.003   0.718   0.279
E S  5CYKA                 98    0.360  42.239  -1.666   0.005   0.336   0.660
B L  5CYKA                 99    0.197  36.052  -0.389   0.004   0.085   0.910
E I  5CYKA                100    0.630 116.476  -1.467   0.005   0.045   0.951
E P  5CYKA                101    0.642  91.100  -1.115   0.005   0.015   0.979
E P  5CYKA                102    0.363  51.566  -0.413   0.018   0.047   0.935
B V  5CYKA                103    0.262  40.331   0.147   0.004   0.420   0.576
B Y  5CYKA                104    0.192  41.009   1.131   0.003   0.718   0.279
B E  5CYKA                105    0.032   5.573  -0.086   0.002   0.816   0.182
B A  5CYKA                106    0.031   3.449  -0.495   0.001   0.900   0.099
B S  5CYKA                107    0.040   4.688  -1.731   0.002   0.816   0.182
B F  5CYKA                108    0.058  11.701  -0.486   0.004   0.616   0.381
B E  5CYKA                109    0.079  13.801  -0.355   0.004   0.197   0.799
E H  5CYKA                110    0.397  72.214  -0.047   0.005   0.045   0.951
E D  5CYKA                111    0.270  38.950  -0.793   0.005   0.015   0.979
E D  5CYKA                112    0.259  37.380  -0.015   0.005   0.045   0.951
B W  5CYKA                113    0.100  23.954   0.485   0.003   0.718   0.279
B V  5CYKA                114    0.018   2.782   1.285   0.001   0.900   0.099
B S  5CYKA                115    0.093  10.923   1.344   0.001   0.900   0.099
B A  5CYKA                116    0.026   2.887   0.040   0.011   0.918   0.071
B V  5CYKA                117    0.052   7.946   0.603   0.002   0.816   0.182
B D  5CYKA                118    0.234  33.734   0.534   0.004   0.514   0.481
B V  5CYKA                119    0.130  20.042   0.033   0.019   0.141   0.840
E L  5CYKA                120    0.700 128.170   0.143   0.018   0.047   0.935
E S  5CYKA                121    0.578  67.730  -0.489   0.018   0.019   0.964
E A  5CYKA                122    0.495  54.516  -0.738   0.018   0.019   0.964
E T  5CYKA                123    0.513  71.181   0.302   0.005   0.262   0.733
E S  5CYKA                124    0.308  36.133   0.891   0.003   0.718   0.279
E P  5CYKA                125    0.320  45.351   1.002   0.018   0.846   0.136
E A  5CYKA                126    0.375  41.314   0.761   0.021   0.756   0.223
B G  5CYKA                127    0.103   8.082   0.266   0.023   0.655   0.322
E R  5CYKA                128    0.574 131.515   0.710   0.004   0.514   0.481
E W  5CYKA                129    0.308  74.050  -1.440   0.004   0.197   0.799
B S  5CYKA                130    0.198  23.170  -0.285   0.004   0.085   0.910
E S  5CYKA                131    0.640  74.961  -0.621   0.005   0.045   0.951
E A  5CYKA                132    0.421  46.361  -0.976   0.005   0.045   0.951
E A  5CYKA                133    0.274  30.195   1.119   0.005   0.262   0.733
B N  5CYKA                134    0.019   2.840   0.772   0.002   0.816   0.182
B S  5CYKA                135    0.155  18.178   1.077   0.001   0.900   0.099
B S  5CYKA                136    0.030   3.516   0.062   0.001   0.959   0.040
B A  5CYKA                137    0.022   2.457   1.072   0.001   0.959   0.040
B A  5CYKA                138    0.076   8.331  -0.403   0.001   0.959   0.040
B V  5CYKA                139    0.051   7.885   0.318   0.001   0.900   0.099
B Q  5CYKA                140    0.093  16.592   0.490   0.004   0.085   0.910
E P  5CYKA                141    0.521  73.930  -0.044   0.005   0.045   0.951
E G  5CYKA                142    0.594  46.771   0.036   0.003   0.003   0.994
E Q  5CYKA                143    0.298  53.187  -0.463   0.003   0.003   0.994
E E  5CYKA                144    0.473  82.668   1.399   0.018   0.047   0.935
B R  5CYKA                145    0.146  33.365   1.932   0.002   0.816   0.182
B V  5CYKA                146    0.019   2.874   1.496   0.011   0.918   0.071
B L  5CYKA                147    0.026   4.687   0.640   0.011   0.918   0.071
B S  5CYKA                148    0.026   3.071  -0.397   0.011   0.918   0.071
B A  5CYKA                149    0.025   2.755  -0.265   0.003   0.718   0.279
B S  5CYKA                150    0.041   4.782  -1.383   0.005   0.336   0.660
E Y  5CYKA                151    0.285  60.840  -0.487   0.005   0.045   0.951
B D  5CYKA                152    0.173  24.886  -1.511   0.005   0.015   0.979
B G  5CYKA                153    0.167  13.111  -0.518   0.005   0.045   0.951
B L  5CYKA                154    0.058  10.565   0.001   0.003   0.718   0.279
B L  5CYKA                155    0.017   3.039   1.544   0.001   0.900   0.099
B R  5CYKA                156    0.065  14.885   1.369   0.001   0.959   0.040
B I  5CYKA                157    0.018   3.367   1.161   0.001   0.959   0.040
B W  5CYKA                158    0.038   9.163   0.539   0.001   0.900   0.099
B N  5CYKA                159    0.223  32.589   0.597   0.003   0.718   0.279
B A  5CYKA                160    0.090   9.951   0.861   0.005   0.262   0.733
E S  5CYKA                161    0.637  74.598   1.032   0.004   0.197   0.799
E G  5CYKA                162    0.522  41.058  -0.409   0.018   0.019   0.964
E S  5CYKA                163    0.498  58.412  -1.004   0.018   0.019   0.964
E V  5CYKA                164    0.596  91.605  -0.192   0.019   0.141   0.840
E I  5CYKA                165    0.310  57.387   0.079   0.022   0.359   0.619
E A  5CYKA                166    0.475  52.323  -0.271   0.022   0.359   0.619
E T  5CYKA                167    0.365  50.639  -0.433   0.022   0.359   0.619
E S  5CYKA                168    0.467  54.709   0.027   0.022   0.359   0.619
E P  5CYKA                169    0.342  48.487  -0.259   0.021   0.451   0.528
E S  5CYKA                170    0.415  48.685  -0.091   0.022   0.552   0.426
E G  5CYKA                171    0.293  23.083   0.341   0.021   0.756   0.223
E S  5CYKA                172    0.434  50.818   0.648   0.023   0.655   0.322
B H  5CYKA                173    0.090  16.316  -0.304   0.021   0.451   0.528
E G  5CYKA                174    0.607  47.787   0.696   0.020   0.205   0.775
E G  5CYKA                175    0.376  29.615  -1.083   0.018   0.047   0.935
B H  5CYKA                176    0.175  31.832  -0.185   0.018   0.047   0.935
E T  5CYKA                177    0.640  88.810  -0.247   0.005   0.045   0.951
E A  5CYKA                178    0.354  39.044  -0.723   0.004   0.085   0.910
B S  5CYKA                179    0.233  27.319   1.018   0.005   0.336   0.660
B I  5CYKA                180    0.018   3.274   1.009   0.001   0.900   0.099
B K  5CYKA                181    0.162  33.323   1.065   0.001   0.959   0.040
B A  5CYKA                182    0.032   3.581  -0.181   0.001   0.959   0.040
B A  5CYKA                183    0.017   1.895   1.453   0.001   0.959   0.040
B K  5CYKA                184    0.087  17.978  -0.124   0.001   0.959   0.040
B F  5CYKA                185    0.058  11.721   0.447   0.002   0.816   0.182
B L  5CYKA                186    0.091  16.644   0.634   0.004   0.085   0.910
E T  5CYKA                187    0.485  67.339  -0.192   0.005   0.015   0.979
E S  5CYKA                188    0.690  80.856   0.188   0.003   0.003   0.994
B D  5CYKA                189    0.247  35.636  -0.410   0.005   0.015   0.979
B R  5CYKA                190    0.204  46.785   1.210   0.004   0.616   0.381
B L  5CYKA                191    0.030   5.475   0.500   0.001   0.900   0.099
B A  5CYKA                192    0.025   2.700   0.938   0.011   0.918   0.071
B S  5CYKA                193    0.029   3.352  -0.432   0.011   0.918   0.071
B A  5CYKA                194    0.031   3.416  -0.688   0.003   0.718   0.279
B G  5CYKA                195    0.055   4.336  -1.087   0.005   0.336   0.660
E M  5CYKA                196    0.303  60.550  -0.223   0.005   0.015   0.979
E D  5CYKA                197    0.287  41.342  -0.680   0.003   0.003   0.994
B R  5CYKA                198    0.177  40.487  -0.085   0.005   0.045   0.951
B T  5CYKA                199    0.073  10.083   0.426   0.003   0.718   0.279
B V  5CYKA                200    0.016   2.521   1.463   0.001   0.900   0.099
B R  5CYKA                201    0.063  14.473   1.402   0.001   0.959   0.040
B V  5CYKA                202    0.019   2.905   1.120   0.001   0.959   0.040
B W  5CYKA                203    0.031   7.576   0.477   0.001   0.900   0.099
B K  5CYKA                204    0.211  43.464   0.529   0.003   0.718   0.279
B Y  5CYKA                205    0.089  19.083   0.903   0.004   0.420   0.576
E T  5CYKA                206    0.576  79.863   0.730   0.005   0.262   0.733
E E  5CYKA                207    0.450  78.563  -0.327   0.018   0.047   0.935
E S  5CYKA                208    0.568  66.581  -1.343   0.018   0.019   0.964
E D  5CYKA                209    0.553  79.658  -0.765   0.052   0.084   0.864
E H  5CYKA                210    0.445  80.945  -0.471   0.056   0.142   0.802
E F  5CYKA                211    0.454  91.138  -0.797   0.056   0.142   0.802
E T  5CYKA                212    0.476  66.063  -0.882   0.052   0.084   0.864
E G  5CYKA                213    0.432  33.998  -1.400   0.056   0.142   0.802
E E  5CYKA                214    0.590 103.073  -0.102   0.022   0.359   0.619
B L  5CYKA                215    0.233  42.571  -0.810   0.004   0.514   0.481
E K  5CYKA                216    0.461  94.848   0.303   0.003   0.718   0.279
E P  5CYKA                217    0.309  43.918   0.170   0.002   0.816   0.182
E T  5CYKA                218    0.376  52.110   0.381   0.002   0.816   0.182
B L  5CYKA                219    0.203  37.078   0.383   0.001   0.900   0.099
E E  5CYKA                220    0.387  67.539   0.763   0.002   0.816   0.182
B L  5CYKA                221    0.067  12.213  -0.252   0.004   0.616   0.381
E Y  5CYKA                222    0.549 117.407   0.827   0.005   0.336   0.660
B G  5CYKA                223    0.238  18.699  -1.387   0.004   0.085   0.910
B H  5CYKA                224    0.179  32.542  -0.441   0.005   0.045   0.951
E T  5CYKA                225    0.637  88.421  -0.342   0.005   0.015   0.979
E G  5CYKA                226    0.371  29.221  -0.989   0.005   0.045   0.951
E S  5CYKA                227    0.248  29.030   1.265   0.004   0.197   0.799
B V  5CYKA                228    0.017   2.613   1.034   0.002   0.816   0.182
B D  5CYKA                229    0.176  25.405   1.140   0.001   0.900   0.099
B W  5CYKA                230    0.036   8.538   0.037   0.001   0.959   0.040
B L  5CYKA                231    0.019   3.406   1.427   0.001   0.959   0.040
B D  5CYKA                232    0.082  11.845   0.325   0.001   0.959   0.040
B V  5CYKA                233    0.048   7.439   0.245   0.001   0.900   0.099
B D  5CYKA                234    0.081  11.629   0.603   0.004   0.197   0.799
E G  5CYKA                235    0.456  35.871  -0.128   0.005   0.045   0.951
E H  5CYKA                236    0.608 110.650   0.489   0.003   0.003   0.994
B S  5CYKA                237    0.247  28.972  -0.536   0.003   0.003   0.994
E K  5CYKA                238    0.438  90.055   1.113   0.005   0.045   0.951
B H  5CYKA                239    0.165  30.032   1.736   0.002   0.816   0.182
B I  5CYKA                240    0.018   3.404   1.484   0.001   0.900   0.099
B L  5CYKA                241    0.029   5.365   0.368   0.011   0.918   0.071
B T  5CYKA                242    0.027   3.689  -0.347   0.001   0.900   0.099
B A  5CYKA                243    0.026   2.865  -0.046   0.002   0.816   0.182
B S  5CYKA                244    0.040   4.688  -1.024   0.004   0.420   0.576
E A  5CYKA                245    0.299  32.895   0.063   0.005   0.015   0.979
B D  5CYKA                246    0.152  21.874  -1.109   0.005   0.015   0.979
B G  5CYKA                247    0.208  16.362   0.068   0.004   0.085   0.910
B A  5CYKA                248    0.059   6.557  -0.140   0.003   0.718   0.279
B I  5CYKA                249    0.020   3.681   1.359   0.001   0.900   0.099
B G  5CYKA                250    0.069   5.407   1.293   0.001   0.959   0.040
B F  5CYKA                251    0.023   4.616   0.825   0.001   0.959   0.040
B W  5CYKA                252    0.040   9.572   0.742   0.001   0.900   0.099
B S  5CYKA                253    0.240  28.140   0.907   0.003   0.718   0.279
B A  5CYKA                254    0.089   9.841   0.491   0.004   0.197   0.799
E S  5CYKA                255    0.698  81.817   1.167   0.005   0.045   0.951
E K  5CYKA                256    0.575 118.277   0.726   0.018   0.019   0.964
E A  5CYKA                257    0.394  43.430  -0.537   0.018   0.019   0.964
E S  5CYKA                258    0.570  66.827   0.615   0.021   0.451   0.528
E A  5CYKA                259    0.305  33.600   0.757   0.018   0.846   0.136
E P  5CYKA                260    0.378  53.624   1.053   0.018   0.846   0.136
E E  5CYKA                261    0.441  76.973   0.448   0.021   0.756   0.223
B P  5CYKA                262    0.255  36.170   0.203   0.023   0.655   0.322
E D  5CYKA                263    0.399  57.554  -0.085   0.022   0.359   0.619
E A  5CYKA                264    0.500  55.078  -0.920   0.004   0.138   0.858
B S  5CYKA                265    0.230  26.956  -0.915   0.018   0.088   0.893
E L  5CYKA                266    0.579 106.070  -0.973   0.018   0.088   0.893
E L  5CYKA                267    0.449  82.230  -1.149   0.004   0.085   0.910
E P  5CYKA                268    0.290  41.194   0.767   0.004   0.197   0.799
B G  5CYKA                269    0.025   1.936   0.053   0.003   0.718   0.279
B A  5CYKA                270    0.218  24.079   0.485   0.001   0.900   0.099
B H  5CYKA                271    0.048   8.713  -0.011   0.001   0.900   0.099
B V  5CYKA                272    0.023   3.458   0.871   0.001   0.959   0.040
B S  5CYKA                273    0.088  10.278  -0.005   0.001   0.959   0.040
B K  5CYKA                274    0.068  14.008  -0.237   0.001   0.900   0.099
B R  5CYKA                275    0.088  20.244   0.392   0.004   0.138   0.858
E R  5CYKA                276    0.532 121.736  -0.169   0.005   0.015   0.979
E K  5CYKA                277    0.595 122.350   0.102   0.003   0.003   0.994
E A  5CYKA                278    0.277  30.481  -0.807   0.003   0.003   0.994
E T  5CYKA                279    0.477  66.229   1.302   0.004   0.085   0.910
B S  5CYKA                280    0.149  17.463   1.655   0.002   0.816   0.182
B S  5CYKA                281    0.027   3.118   1.070   0.001   0.900   0.099
B V  5CYKA                282    0.024   3.689   0.746   0.011   0.918   0.071
B S  5CYKA                283    0.033   3.926  -1.093   0.001   0.900   0.099
B T  5CYKA                284    0.029   4.008  -0.524   0.003   0.718   0.279
B A  5CYKA                285    0.057   6.314  -1.072   0.005   0.336   0.660
E Q  5CYKA                286    0.345  61.671  -0.181   0.005   0.015   0.979
E R  5CYKA                287    0.368  84.226  -0.738   0.005   0.015   0.979
B G  5CYKA                288    0.208  16.338   0.114   0.005   0.045   0.951
B P  5CYKA                289    0.085  12.047   0.551   0.004   0.616   0.381
B L  5CYKA                290    0.017   3.076   1.461   0.001   0.900   0.099
B G  5CYKA                291    0.100   7.862   1.277   0.001   0.900   0.099
B L  5CYKA                292    0.030   5.548   0.227   0.011   0.918   0.071
B W  5CYKA                293    0.051  12.265   0.689   0.002   0.816   0.182
E S  5CYKA                294    0.252  29.488   0.168   0.004   0.514   0.481
B I  5CYKA                295    0.138  25.438  -0.853   0.018   0.088   0.893
B H  5CYKA                296    0.248  45.075  -0.427   0.004   0.085   0.910
E T  5CYKA                297    0.573  79.447   0.148   0.005   0.045   0.951
E A  5CYKA                298    0.462  50.868  -0.738   0.005   0.015   0.979
E P  5CYKA                299    0.376  53.354   1.107   0.004   0.197   0.799
B A  5CYKA                300    0.020   2.259   0.819   0.002   0.816   0.182
B T  5CYKA                301    0.205  28.489   0.774   0.001   0.900   0.099
B A  5CYKA                302    0.036   4.000   0.012   0.001   0.900   0.099
B A  5CYKA                303    0.019   2.050   1.291   0.001   0.959   0.040
B I  5CYKA                304    0.071  13.191   0.031   0.001   0.959   0.040
B F  5CYKA                305    0.050  10.115   0.108   0.002   0.816   0.182
B D  5CYKA                306    0.071  10.246   0.432   0.004   0.138   0.858
E P  5CYKA                307    0.427  60.549  -0.305   0.005   0.045   0.951
E R  5CYKA                308    0.597 136.599   0.155   0.003   0.003   0.994
E D  5CYKA                309    0.360  51.934  -1.436   0.003   0.003   0.994
E R  5CYKA                310    0.452 103.600   0.757   0.005   0.045   0.951
B T  5CYKA                311    0.218  30.264   1.456   0.003   0.718   0.279
B V  5CYKA                312    0.034   5.303   0.930   0.001   0.900   0.099
B A  5CYKA                313    0.025   2.766   0.926   0.011   0.918   0.071
B Y  5CYKA                314    0.054  11.476   0.073   0.001   0.959   0.040
B S  5CYKA                315    0.022   2.602   0.037   0.001   0.959   0.040
B A  5CYKA                316    0.030   3.306  -0.623   0.003   0.718   0.279
B S  5CYKA                317    0.053   6.165  -1.022   0.004   0.197   0.799
E Q  5CYKA                318    0.321  57.348   0.085   0.005   0.015   0.979
B D  5CYKA                319    0.213  30.693  -1.332   0.005   0.015   0.979
B H  5CYKA                320    0.134  24.375   0.291   0.004   0.085   0.910
B T  5CYKA                321    0.066   9.113  -0.077   0.003   0.718   0.279
B V  5CYKA                322    0.018   2.736   1.520   0.001   0.900   0.099
B R  5CYKA                323    0.068  15.480   1.174   0.001   0.959   0.040
B T  5CYKA                324    0.027   3.787   0.343   0.001   0.900   0.099
B L  5CYKA                325    0.041   7.434   0.499   0.001   0.900   0.099
E D  5CYKA                326    0.276  39.829   0.676   0.003   0.718   0.279
B L  5CYKA                327    0.071  12.963   0.286   0.019   0.141   0.840
E T  5CYKA                328    0.616  85.370   0.637   0.018   0.047   0.935
E T  5CYKA                329    0.594  82.388   0.844   0.018   0.019   0.964
E G  5CYKA                330    0.347  27.270  -0.140   0.018   0.019   0.964
E Q  5CYKA                331    0.588 105.035   0.779   0.021   0.279   0.699
E V  5CYKA                332    0.280  43.067   0.961   0.021   0.756   0.223
E V  5CYKA                333    0.370  56.869   1.086   0.018   0.846   0.136
E S  5CYKA                334    0.261  30.542   1.341   0.011   0.918   0.071
E T  5CYKA                335    0.423  58.656   1.382   0.011   0.918   0.071
B L  5CYKA                336    0.080  14.648   0.716   0.003   0.718   0.279
E T  5CYKA                337    0.608  84.288   1.084   0.004   0.514   0.481
B L  5CYKA                338    0.256  46.782  -1.056   0.004   0.138   0.858
B T  5CYKA                339    0.235  32.581  -0.286   0.018   0.047   0.935
E H  5CYKA                340    0.656 119.254  -0.066   0.018   0.047   0.935
E P  5CYKA                341    0.479  67.942  -0.560   0.005   0.045   0.951
E L  5CYKA                342    0.288  52.751   1.360   0.005   0.262   0.733
B L  5CYKA                343    0.026   4.724   0.219   0.002   0.816   0.182
B S  5CYKA                344    0.153  17.896   1.134   0.001   0.900   0.099
B L  5CYKA                345    0.026   4.852   0.317   0.001   0.959   0.040
B S  5CYKA                346    0.015   1.699   1.537   0.001   0.959   0.040
B A  5CYKA                347    0.040   4.386  -0.351   0.001   0.959   0.040
B L  5CYKA                348    0.029   5.383   0.463   0.001   0.900   0.099
B T  5CYKA                349    0.054   7.504   0.649   0.004   0.420   0.576
B R  5CYKA                350    0.180  41.312  -1.054   0.004   0.197   0.799
E A  5CYKA                351    0.473  52.169  -0.521   0.005   0.015   0.979
B G  5CYKA                352    0.267  21.044  -1.875   0.003   0.003   0.994
E T  5CYKA                353    0.524  72.707  -0.887   0.018   0.019   0.964
E T  5CYKA                354    0.519  71.916  -0.373   0.004   0.085   0.910
E S  5CYKA                355    0.354  41.465  -0.005   0.004   0.085   0.910
E P  5CYKA                356    0.316  44.812   0.930   0.005   0.336   0.660
B L  5CYKA                357    0.084  15.454   1.407   0.002   0.816   0.182
B L  5CYKA                358    0.014   2.545   1.699   0.001   0.900   0.099
B A  5CYKA                359    0.019   2.116   0.992   0.001   0.959   0.040
B A  5CYKA                360    0.020   2.193   0.324   0.001   0.900   0.099
B G  5CYKA                361    0.025   1.928   0.002   0.003   0.718   0.279
B T  5CYKA                362    0.045   6.283  -0.613   0.005   0.262   0.733
E S  5CYKA                363    0.337  39.473  -0.239   0.005   0.015   0.979
B A  5CYKA                364    0.182  20.023  -1.206   0.003   0.003   0.994
B R  5CYKA                365    0.192  44.014  -0.074   0.005   0.045   0.951
B H  5CYKA                366    0.069  12.569   0.587   0.004   0.616   0.381
B I  5CYKA                367    0.016   2.923   1.592   0.001   0.900   0.099
B T  5CYKA                368    0.058   8.086   1.550   0.001   0.959   0.040
B M  5CYKA                369    0.019   3.862   1.145   0.001   0.959   0.040
B V  5CYKA                370    0.040   6.087   0.657   0.001   0.900   0.099
B D  5CYKA                371    0.161  23.258   0.245   0.003   0.718   0.279
B P  5CYKA                372    0.087  12.303   0.562   0.004   0.197   0.799
E R  5CYKA                373    0.605 138.591   0.647   0.004   0.138   0.858
E A  5CYKA                374    0.479  52.808   0.076   0.018   0.047   0.935
E S  5CYKA                375    0.349  40.856  -1.221   0.058   0.017   0.925
E S  5CYKA                376    0.670  78.465   0.106   0.053   0.043   0.903
E A  5CYKA                377    0.476  52.499  -0.220   0.052   0.084   0.864
E T  5CYKA                378    0.384  53.289  -0.540   0.064   0.216   0.721
E T  5CYKA                379    0.466  64.593  -0.116   0.066   0.296   0.638
E S  5CYKA                380    0.334  39.121   0.446   0.074   0.484   0.442
B V  5CYKA                381    0.203  31.170   0.267   0.022   0.552   0.426
E M  5CYKA                382    0.331  66.333   0.524   0.023   0.655   0.322
E T  5CYKA                383    0.405  56.215   1.131   0.023   0.655   0.322
B L  5CYKA                384    0.080  14.575  -0.109   0.022   0.552   0.426
E R  5CYKA                385    0.568 130.003   0.672   0.022   0.359   0.619
E G  5CYKA                386    0.308  24.232  -0.905   0.019   0.141   0.840
B H  5CYKA                387    0.172  31.269  -0.221   0.018   0.047   0.935
E A  5CYKA                388    0.640  70.484  -0.240   0.018   0.047   0.935
E N  5CYKA                389    0.438  64.138  -0.782   0.005   0.045   0.951
B K  5CYKA                390    0.220  45.151   1.188   0.004   0.197   0.799
B V  5CYKA                391    0.018   2.813   0.886   0.002   0.816   0.182
B V  5CYKA                392    0.149  22.886   0.671   0.001   0.959   0.040
B S  5CYKA                393    0.037   4.290   0.283   0.001   0.959   0.040
B L  5CYKA                394    0.016   2.856   1.549   0.001   0.959   0.040
B S  5CYKA                395    0.054   6.317  -0.024   0.001   0.959   0.040
B P  5CYKA                396    0.036   5.037  -0.119   0.001   0.900   0.099
B S  5CYKA                397    0.064   7.501   0.480   0.005   0.262   0.733
E P  5CYKA                398    0.332  47.068  -0.108   0.004   0.085   0.910
E E  5CYKA                399    0.590 103.090   0.005   0.005   0.015   0.979
E N  5CYKA                400    0.369  53.963  -1.001   0.003   0.003   0.994
E E  5CYKA                401    0.544  94.967   1.098   0.005   0.015   0.979
B Y  5CYKA                402    0.202  43.125   0.852   0.004   0.616   0.381
B S  5CYKA                403    0.082   9.646   1.387   0.002   0.816   0.182
B L  5CYKA                404    0.017   3.039   1.437   0.001   0.900   0.099
B V  5CYKA                405    0.025   3.796   0.668   0.001   0.959   0.040
B S  5CYKA                406    0.022   2.625   0.157   0.001   0.900   0.099
B G  5CYKA                407    0.032   2.558  -0.690   0.002   0.816   0.182
B S  5CYKA                408    0.052   6.141  -0.641   0.005   0.262   0.733
E H  5CYKA                409    0.311  56.516  -0.529   0.005   0.015   0.979
E D  5CYKA                410    0.229  33.042  -0.979   0.005   0.015   0.979
B G  5CYKA                411    0.167  13.111  -0.168   0.005   0.045   0.951
B T  5CYKA                412    0.071   9.834   0.305   0.003   0.718   0.279
B C  5CYKA                413    0.019   2.682   1.454   0.001   0.900   0.099
B R  5CYKA                414    0.075  17.221   1.314   0.001   0.959   0.040
B V  5CYKA                415    0.024   3.627   0.703   0.001   0.900   0.099
B W  5CYKA                416    0.041   9.909   0.603   0.001   0.900   0.099
B D  5CYKA                417    0.234  33.719   0.244   0.003   0.718   0.279
B L  5CYKA                418    0.083  15.252   0.485   0.019   0.141   0.840
E R  5CYKA                419    0.638 146.194   0.515   0.018   0.047   0.935
E S  5CYKA                420    0.566  66.370   0.387   0.018   0.019   0.964
E V  5CYKA                421    0.353  54.195   0.069   0.018   0.047   0.935
E R  5CYKA                422    0.589 134.789   0.855   0.022   0.359   0.619
E P  5CYKA                423    0.288  40.810   0.981   0.021   0.756   0.223
E A  5CYKA                424    0.384  42.339   0.550   0.018   0.846   0.136
E T  5CYKA                425    0.301  41.804   0.741   0.018   0.846   0.136
E K  5CYKA                426    0.460  94.601   1.166   0.021   0.756   0.223
B E  5CYKA                427    0.076  13.312   0.101   0.004   0.616   0.381
E E  5CYKA                428    0.662 115.582   0.920   0.005   0.336   0.660
E G  5CYKA                429    0.377  29.678  -0.791   0.004   0.085   0.910
B S  5CYKA                430    0.236  27.612  -0.558   0.018   0.047   0.935
E L  5CYKA                431    0.682 124.856  -0.188   0.018   0.047   0.935
E G  5CYKA                432    0.439  34.581  -0.782   0.004   0.085   0.910
E G  5CYKA                433    0.258  20.320   1.157   0.005   0.262   0.733
B V  5CYKA                434    0.022   3.305   0.430   0.002   0.816   0.182
B S  5CYKA                435    0.166  19.479   1.045   0.001   0.900   0.099
B E  5CYKA                436    0.051   8.840  -0.020   0.001   0.959   0.040
B P  5CYKA                437    0.025   3.576   0.829   0.001   0.959   0.040
B V  5CYKA                438    0.068  10.390  -0.352   0.001   0.959   0.040
B Y  5CYKA                439    0.075  16.070  -0.116   0.002   0.816   0.182
B V  5CYKA                440    0.069  10.682   0.445   0.004   0.138   0.858
E I  5CYKA                441    0.470  86.876  -0.105   0.005   0.045   0.951
E E  5CYKA                442    0.647 113.101  -0.129   0.003   0.003   0.994
E R  5CYKA                443    0.283  64.784  -0.887   0.018   0.019   0.964
E E  5CYKA                444    0.433  75.732   0.209   0.021   0.279   0.699
B S  5CYKA                445    0.207  24.296   1.053   0.018   0.846   0.136
B W  5CYKA                446    0.074  17.869   0.335   0.011   0.918   0.071
B A  5CYKA                447    0.070   7.670  -0.682   0.018   0.846   0.136
B S  5CYKA                448    0.102  11.908  -1.662   0.023   0.655   0.322
B K  5CYKA                449    0.184  37.849  -1.745   0.021   0.451   0.528
B G  5CYKA                450    0.196  15.386  -1.720   0.020   0.205   0.775
E K  5CYKA                451    0.505 103.837  -0.069   0.020   0.205   0.775
E K  5CYKA                452    0.346  71.193  -0.487   0.022   0.359   0.619
E K  5CYKA                453    0.342  70.411  -0.054   0.022   0.359   0.619
E R  5CYKA                454    0.421  96.409   0.163   0.022   0.552   0.426
E P  5CYKA                455    0.383  54.376   0.637   0.022   0.552   0.426
B V  5CYKA                456    0.119  18.244   0.021   0.021   0.451   0.528
E A  5CYKA                457    0.564  62.109   0.401   0.022   0.359   0.619
E G  5CYKA                458    0.412  32.409  -1.261   0.019   0.141   0.840
E D  5CYKA                459    0.285  41.083  -0.494   0.018   0.088   0.893
E G  5CYKA                460    0.669  52.642  -0.806   0.018   0.047   0.935
E C  5CYKA                461    0.436  61.158  -1.058   0.005   0.045   0.951
E K  5CYKA                462    0.316  64.919   1.023   0.004   0.197   0.799
B V  5CYKA                463    0.034   5.272  -0.622   0.003   0.718   0.279
B F  5CYKA                464    0.162  32.533   0.667   0.002   0.816   0.182
B S  5CYKA                465    0.056   6.610  -0.046   0.001   0.900   0.099
B V  5CYKA                466    0.032   4.872  -0.075   0.001   0.900   0.099
B V  5CYKA                467    0.115  17.614  -0.741   0.002   0.816   0.182
B W  5CYKA                468    0.102  24.603  -0.313   0.004   0.616   0.381
B D  5CYKA                469    0.144  20.779   0.361   0.005   0.045   0.951
E K  5CYKA                470    0.535 109.988  -0.916   0.003   0.003   0.994
E L  5CYKA                471    0.466  85.398  -0.393   0.005   0.015   0.979
B G  5CYKA                472    0.084   6.595   0.536   0.004   0.616   0.381
B I  5CYKA                473    0.022   4.070   0.707   0.002   0.816   0.182
B F  5CYKA                474    0.027   5.339   0.452   0.011   0.918   0.071
B S  5CYKA                475    0.024   2.789   0.021   0.011   0.918   0.071
B G  5CYKA                476    0.028   2.227  -0.207   0.003   0.718   0.279
B G  5CYKA                477    0.049   3.864  -0.537   0.004   0.420   0.576
E E  5CYKA                478    0.308  53.877  -0.114   0.005   0.045   0.951
B D  5CYKA                479    0.225  32.408  -0.871   0.005   0.015   0.979
B K  5CYKA                480    0.174  35.854   0.044   0.004   0.138   0.858
B K  5CYKA                481    0.094  19.356   0.852   0.002   0.816   0.182
B V  5CYKA                482    0.020   3.089   1.385   0.001   0.959   0.040
B Q  5CYKA                483    0.079  14.038   1.626   0.001   0.959   0.040
B V  5CYKA                484    0.024   3.689   0.590   0.001   0.900   0.099
B N  5CYKA                485    0.058   8.520   0.587   0.001   0.900   0.099
E R  5CYKA                486    0.370  84.822   1.549   0.023   0.655   0.322
B G  5CYKA                487    0.124   9.743  -0.316   0.020   0.205   0.775
E R  5CYKA                488    0.702 160.781   1.001   0.064   0.216   0.721
E N  5CYKA                489    0.553  80.959  -0.137   0.052   0.084   0.864
E I  5CYKA                490    0.332  61.365   0.235   0.052   0.084   0.864
E V  5CYKA                491    0.539  82.860   0.955   0.069   0.386   0.545
E T  5CYKA                492    0.416  57.630   0.652   0.074   0.484   0.442
E E  5CYKA                493    0.574 100.348   0.697   0.069   0.386   0.545
E Q  5CYKA                494    0.450  80.388  -0.097   0.066   0.296   0.638
E K  5CYKA                495    0.719 147.939   1.242   0.005   0.015   0.979

Explain the output.  Go back.
```

---
